# Supplementary material for: Beyond Hypoglossal Hype: Social Media Perspectives on the Inspire Upper Airway Stimulation System
Source: Healthcare (Basel). 2023 Dec 1;11(23):3082. doi: 10.3390/healthcare11233082 (PMC10706326; doi:10.3390/healthcare11233082)
Supplement: Supplementary file 1 [file healthcare-11-03082-s001.zip › healthcare-2730458-supplementary.pdf]

Inspire® Upper Airway Stimulation (UAS) Social Media Analysis

Data Collection Guidelines

Objective: To systematically collect, categorize, and analyze social media posts related to Inspire® Upper Airway Stimulation (UAS) across Instagram, Facebook, and TikTok.

Search Terms:
- #inspiresleep, #inspiresleepapnea, #inspiresleepapneaimplant, #hypoglossalnervestimulator, #lifewithinspire.
- Record and categorize hashtags. Assess the frequency and context in which each hashtag is used.

Detailed Data Collection Categories

Platform Identification
- **Platforms**: Instagram, Facebook, TikTok.
- **Procedure**: Record the platform where the post is found.

Post Type
- **Categories**: Image, Video, Text.
- **Procedure**: Classify based on the primary media type.

Authorship Classification
- **Categories**:
    - Inspire® Company
    - Patient
    - Patient’s Family
    - Physicians
    - Non-Physician Healthcare Providers
    - Media Outlet
    - Professional Organization
    - Academic Institution
- **Procedure**: Determine the author based on profile information and affiliations. For instance, a post from a profile clearly identified as a doctor’s personal or professional account should be categorized under 'Physicians'.

Subject Matter Categorization
- **Categories**: Advertisement, Educational, Patient Experience, Media Coverage
- **Procedure**:
    - *Advertisement*: Includes promotional content, marketing materials, or posts aimed at selling or endorsing the Inspire® device or related services
    - *Educational*: Contains informative content about UAS, procedural details, or general sleep apnea awareness

- *Patient Experience*: Shares personal stories, testimonials, or patient journey narratives
- *Media Coverage*: Involves news articles, media features, or documentaries about UAS

- **Guidelines**: Evaluate the main message or focus of the post. If it primarily serves to educate, classify it as 'Educational'. If it seems aimed at promoting a product or service, categorize it as 'Advertisement'.

Popularity Measurement

- **Metric**: Number of likes on individual post
- **Procedure**: Record the total likes a post has received at the time of data collection.

Date and Timeframe Documentation

- **Format**: Record the year and the exact date (dd/mm/yyyy) of the post.

Engagement Analysis

- **Metrics**: Likes, comments, shares.
- **Procedure**: Record all relevant engagement metrics available for each post.

Content Tone and Sentiment Analysis

- **Categories**: Positive, Negative, Neutral.
- **Procedure**: Assess the overall sentiment of the post based on language, emojis, and context.

***Ethical Considerations*:**

- Ensure that all data is collected from publicly available sources
- Maintain neutrality and avoid introducing personal biases during data collection and categorization
- Respect user privacy and platform-specific data use policies

***Notes for Data Collectors*:**

- Accurate categorization is crucial for the integrity of the study
- If a post's category is unclear, reviewers should discuss and reach a consensus
- Training sessions and periodic meetings can help maintain consistency in data collection and interpretation
